# Supplementary material for: Burden among informal caregivers of individuals with heart failure: A mixed methods study
Source: PLoS One. 2023 Nov 17;18(11):e0292948. doi: 10.1371/journal.pone.0292948 (PMC10656022; doi:10.1371/journal.pone.0292948)
Supplement: S1 Checklist — (DOCX) [file pone.0292948.s001.docx]

Good Reporting of A Mixed Methods Study (GRAMMS) checklist

| **Guideline** | **Section: page** |
| --- | --- |
| Describe the justification for using a mixed methods approach to the research question | Introduction and Methods (design) pag. 2 |
| Describe the design in terms of the purpose, priority and sequence of methods | Methods (design) pag. 2 |
| Describe each method in terms of sampling, data collection and analysis | Quant:  Sampling → Methods (setting and sampling) pag. 3  Data collection → Methods (data collection) pag. 4  Data analysis → Methods (data analysis) pag. 5  Qual:    Sampling → Methods (setting and sampling) pag. 4  Data collection → Methods (data collection) pag. 4  Data analysis → Methods (data analysis) pag. 5 |
| Describe where integration has occurred, how it has occurred and who has participated in it | Results → Mixed methods analysis pag. 10 |
| Describe any limitation of one method associated with the present of the other method | Limitations and Strengths → pag. 12 |
| Describe any insights gained from mixing or integrating methods | Discussions → pag. 11 |

O'Cathain A, Murphy E, Nicholl J. The quality of mixed methods studies in health services research. J Health Serv Res Policy. 2008;13: 92-98.
