# Supplementary material for: Burden among informal caregivers of individuals with heart failure: A mixed methods study
Source: PLoS One. 2023 Nov 17;18(11):e0292948. doi: 10.1371/journal.pone.0292948 (PMC10656022; doi:10.1371/journal.pone.0292948)
Supplement: Attachment — Submitted filename: Authors Response.docx [file pone.0292948.s003.docx]

| **Journal requirements** | |
| --- | --- |
| 1. Please ensure that your manuscript meets PLOS ONE's style requirements, including those for file naming.   ThePLOS ONE style templates can be found at:  https://journals.plos.org/plosone/s/file?id=wjVg/PLOSOne_formatting_sample_main_body.pdf and  https://journals.plos.org/plosone/s/file?id=ba62/PLOSOne_formatting_sample_title_authors_affiliations.pdf | Thank you for the suggestion. We checked our files and ensured that our manuscript meets PLOS ONE’s style. |
| 2. We note that the grant information you provided in the ‘Funding Information’ and ‘Financial Disclosure’ sections do not match. When you resubmit, please ensure that you provide the correct grant numbers for the awards you received for your study in the ‘Funding Information’ section. | Thanks for the feedback we will report the complete and correct information in the cover letter.  A copy of the correct statement is reported below.  “The first author was funded by the Heart Failure Association with the Nursing Training Fellowship Award. The project received funding also from the CECRI Centre of Excellence for Nursing Scholarship in Rome (2.12.5).” |
| 3. Thank you for stating the following in the Acknowledgments Section of your manuscript:   “The first author was funded by the Heart Failure Association with the Nursing Training Fellowship Award. The project received funding also from the CECRI Centre of Excellence for Nursing Scholarship in Rome (2.12.5).”  We note that you have provided funding information that is not currently declared in your Funding Statement. However, funding information should not appear in the Acknowledgments section or other areas of your manuscript. We will only publish funding information present in the Funding Statement section of the online submission form.  Please remove any funding-related text from the manuscript and let us know how you would like to update your Funding Statement. Currently, your Funding Statement reads as follows:  “The funders had no role in study design, data collection and analysis, decision to publish, or preparation of the manuscript.”  Please include your amended statements within your cover letter; we will change the online submission form on your behalf. | Thank you for noting this. We have corrected this as recommended. Please use the following funding statement.  “The first author was funded by the Heart Failure Association with the Nursing Training Fellowship Award. The project received funding also from the CECRI Centre of Excellence for Nursing Scholarship in Rome (2.12.5).” |
| 4. We note that you have indicated that data from this study are available upon request. PLOS only allows data to be available upon request if there are legal or ethical restrictions on sharing data publicly. For more information on unacceptable data access restrictions, please see http://journals.plos.org/plosone/s/data-availability#loc-unacceptable-data-access-restrictions.   In your revised cover letter, please address the following prompts:  a) If there are ethical or legal restrictions on sharing a de-identified data set, please explain them in detail (e.g., data contain potentially sensitive information, data are owned by a third-party organization, etc.) and who has imposed them (e.g., an ethics committee). Please also provide contact information for a data access committee, ethics committee, or other institutional body to which data requests may be sent.  b) If there are no restrictions, please upload the minimal anonymized data set necessary to replicate your study findings as either Supporting Information files or to a stable, public repository and provide us with the relevant URLs, DOIs, or accession numbers. For a list of acceptable repositories, please see http://journals.plos.org/plosone/s/data-availability#loc-recommended-repositories.  We will update your Data Availability statement on your behalf to reflect the information you provide. | Data supporting the results of this study are available upon reasonable request to the corresponding author, Angela Durante. The data are not publicly available because quantitative and qualitative data are included in the same database due to the mixed nature of the analysis. In particular, the qualitative interviews, given the freedom of expression of the participants, contain information that could compromise the privacy of the research participants or the settings where the interviews were conducted. Furthermore, places and hospital facilities were mentioned in interviews. Sharing that information could easily lead back to the identity of the participants and therefore not respecting data confidentiality. |

| **Reviewers' comments** | |
| --- | --- |
| **Reviewer #1** | |
| Thank you for the opportunity to review “Burden Among Informal Caregivers of Individuals with HeartFailure: A Mixed Methods Study”. The topic is interesting as well as the manuscript itself. Many correlates of caregiver burden are already known, but there is still a lack of qualitative studies exploring the nature of caregiver burden. This article gives an insight into the emotional processes of the dyad and this is its great value. Although the article is well organized there are a few comments that may improve it:I suggest including mutuality, which can be defined as the degree of emotional engagement and mutual support in the dyad, as an additional crucial feature in the Introduction section.(see i.e. Uchmanowicz I, Faulkner KM, Vellone E, Siennicka A, Szczepanowski R, Olchowska-Kotala A. HeartFailure Care: Testing Dyadic Dynamics Using the Actor-Partner Interdependence Model (APIM)-A Scoping Review.Int J Environ Res Public Health. 2022 Feb 9;19(4):1919. doi: 10.3390/ijerph19041919. PMID: 35206131; PMCID:PMC8871794. ; Hooker SA, Schmiege SJ, Trivedi RB, Amoyal NR, Bekelman DB. Mutuality and heart failure self-care in patients and their informal caregivers. Eur J Cardiovasc Nurs. 2018 Feb;17(2):102-113. doi:10.1177/1474515117730184. Epub 2017 Sep 4. PMID: 28868917; PMCID: PMC9390005.)  It would also be advisable to include in the introduction the work of Strömberg (Strömberg, A.; Luttika, M.L. Burdenof Caring: Risks and Consequences Imposed on Caregivers of Those Living and Dyingwith Advanced Heart Failure.Current Opinion in Supportive and Palliative Care. 2015, pp 26–30.https://doi.org/10.1097/SPC.0000000000000111, who draws attention to having an uncertain HF trajectory that places a significant burden on heart failure caregivers. | We are glad that you appreciated our work.  As you suggested we have carefully reviewed the references and have taken appropriate action including these meaningful references to our manuscript in ‘Introduction’ section. Please see the changes performed on page 3 and 4. |
| **Reviewer #2** | |
| Thank you for providing the opportunity to review the manuscript “Burden Among Informal Caregivers of Individuals with Heart Failure: A Mixed Methods Study”. The aim of this study was to develop a comprehensive understanding of caregiver burden and its predictors from a dyadic perspective. The following are the comments.  Introduction:1. It would be better to make some expansion on the concerning variables that have been chosen for exploration alongside caregiver burden. | Thank you for the suggestion. We already report the variables explored in relation to burden on page 4. Although, as you underlined, we agreed with the suggestion of having a focus concerning other variables. Therefore, we included in ‘Data Collection Instruments’ a brief description of each variable mentioned (pages 5 and 6). |
| 2. The author repeatedly emphasized the lack of "Cross-cultural studies" in this area, and the sample was from three different countries. However, cross-cultural differences and analysis were not reflected in the current study | Thank you for highlighting this important issue. Let us preface with the fact that this study is unique as it is one of the very few to include countries in central and southern Europe that are not English-speaking and are countries with different languages, using a mixed method.  We would like to point out that in the quantitative part we performed the analysis and reported the results considering all participants from each country as belonging to a unique sample (European).  Although in the qualitative part, we detected some cultural differences, they were not reported in this study because it was not aligned with the focus of inquiry. In fact, in this work, we highlighted those negative experiences that commonly lead to burden with the scope of trigger critical thinking in the readers to find valid solutions in different contexts based on commonalities instead of differences. Hence, what was in common and not what specifically for each sub-groups was not reported in detail. Cultural differences, in line with the general aim of the project, were reported in the parent study with determinants. |
| 3. Considering the accumulation of a large amount of literature related to the caregiver burden, the description of this sentence “Furthermore, there is a dearth of qualitative studies exploring the nature of caregiver burden and any additional predictors that may affect the caregiver burden” need to be cautious. | Thank you for the suggestion. We formulated this sentence highlighting that no so much *mixed method* studies were performed about burden in caregiving of people with HF.  Please see the variation on page 4. |
| **Methods** | |
| 1. In Data Collection Instruments, please specify in which sample SF-8 was used, patients, caregivers, or both? | Thank you for bringing this to our attention. More text was added to clarify that the questionnaire was administrated to both for caregivers and patients. Please see the variation on page 7. |
| 2. Considering that interview guide of the qualitative phase was not built for the specific purpose declared in this study, it would be better to list a few interview questions for understanding easier. | Thank you for the clarification request. Although we already pointed out that all details were included in the parent study of this second analysis on page 5, we agreed with the reviewer in specifying some of the interview questions. |
| 3. In Setting and Sample, the sample size is 229, it does not match the 184 reported in the results section. How many caregivers are there to participate in the qualitative research? | Thank you for the clarification request. The difference between the number of the entire sample (229) was due to the fact that for perform the analysis we used only those caregivers who did not present any ‘missing’ in answers, hence 184 of 229. This information has been added in data analysis paragraph at page 8.  A nested sample of 50 caregivers participated in semi-structured interviews to better understand the caregiver experience. This information has been added on page 11 at the beginning of the session “Qualitative Findings”. |
| **Results** | |
| 1. In the first paragraph, the description of frequency and percentage is incorrect (cannot use “±” and SD). Additionally, the frequency and percentage of patients' marital status does not match that in the Table1. Please correct it. | Thank you for this important feedback, we are sorry for the inconvenience. We corrected as per your suggestion. Changes on page 9. |
| 2. Qualitative Findings-Repetitive Demands, the summary of this topic does not align with the example listed, please provide a new one to match better. | Thank you for the suggestion, we changed in the text with a more appropriate quotation from caregivers’ interviews.  Please, see changes on page 15. |
| 3. Page 15, line 1-2: the “restricting life” is inconsistent with the topic title “Restructuring Life”. | Thank you for the suggestion. We choose a better quotation for a clearer example. Please see the variation on page 15. |
